# Postural control at posturography with virtual reality in the intercritical period of vestibular migraine

**DOI:** 10.1016/j.bjorl.2019.06.015

**Published:** 2019-08-02

**Authors:** Suelen Cesaroni, Adriana Marques da Silva, Maurício Malavasi Ganança, Heloisa Helena Caovilla

**Affiliations:** a Universidade Federal de São Paulo (Unifesp), Escola Paulista de Medicina (EPM), Programa de Pós-Graduação em Distúrbios da Comunicação Humana, São Paulo, SP, Brazil; b Universidade Federal de São Paulo (Unifesp), Escola Paulista de Medicina (EPM), São Paulo, SP, Brazil; c Universidade Federal de São Paulo (Unifesp), Escola Paulista de Medicina (EPM), Disciplina de Otologia e Otoneurologia, São Paulo, SP, Brazil

**Keywords:** Vestibular migraine, Posturography, Dizziness

## Abstract

**Introduction:**

Vestibular migraine is a condition that associates headache and vestibular symptoms.

**Objective:**

To evaluate body-balance with virtual reality posturography in vestibular migraine.

**Methods:**

A total of 26 patients in the intercritical period of vestibular migraine were compared by means of the Balance Rehabilitation Unit^MT^ (Medical/Interacoustics) posturography with 30 controls, paired for age and gender.

**Results:**

There was no significant statistical difference (*p* = 0.121) in the limit of stability area (cm^2^) between the experimental group and the control group values. There were significant differences (*p* < 0.05) in the values of sway velocity (cm/s) in nine of ten evaluated sensory conditions and in the pressure center displacement area (cm^2^) values in eight of those ten sensory conditions in the comparison between the control group and the experimental group.

**Conclusion:**

Posturography with virtual reality can identify changes in the sway velocity and the pressure center displacement area, characterizing the inability to maintain postural control with and without visual deprivation in situations of visual conflict and vestibulovisual interaction,in the intercritical period of the vestibular migraine.

## Introduction

Vestibular migraine is a clinical condition that associates migraine headache and vestibular symptoms,^1^, ^2^ with relevant effects on quality of life.^3^ Imbalance, postural instability,^1^, ^4^, ^5^ susceptibility to motion sickness, nausea, vomiting, exhaustion and auditory symptoms^2^ can also occur.

Vestibular migraine is estimated to occur in approximately 1% of the general population throughout life.^3^ Prevalence data vary according to the diagnostic criteria and studied populations. It was identified in 11.0% of the patients of a German clinic specializing in dizziness,^6^ in 16.0% of the cases in a Belgian otorhinolaryngological clinic^7^ and in 10.3% of patients in Korean neurological clinics.^8^ The occurrence is higher in the female gender at any age group.^1^

The initially proposed criteria for the diagnosis of vestibular migraine^6^ were used as the basis for the criteria of the Bárány Society and the International Headache Society.^2^, ^9^

The diagnosis of vestibular migraine is based on recurrent vestibular symptoms, such as different types of vertigo or dizziness, with nausea, of moderate or severe intensity, lasting from 5 min to 72 h; history of current or past migraine, with or without aura; temporal association between vestibular symptoms and migraine symptoms; and, exclusion of other causes of vestibular complaints. In approximately 50% of the vestibular episodes, migraine may present with a headache, with at least two of the following characteristics: unilateral, pulsatile, moderate or severe, aggravated by routine physical activities or exercises; and/or with photophobia and phonophobia.^2^

Clinical evaluation and laboratory or functional assessment of patients with vestibular migraine may reveal signs of vestibular dysfunction, which are not pathognomonic of the condition.^4^, ^10^ These findings can identify whether the impairment is peripheral or central^1^ and what strategies are used by these patients to maintain body balance.

Different types of posturography have been used to evaluate the postural control of patients with migraine and vestibular dysfunction, identifying somatosensory, vestibular and visual alterations, isolated or combined,^4^, ^11^, ^12^, ^13^, ^14^, ^15^ but these studies did not use virtual reality stimuli.

Virtual reality is a type of artificial immersion in a safe and controlled environment, which has the ability to expose the patient to sensory conflicts at different levels of difficulty.^16^

In virtual reality, the perception of the environment is modified by the repetitive presentation of visual and proprioceptive stimuli, resulting in sensory conflicts that have an effect on vestibuloocular and vestibulospinal reflexes involved in postural control and in body balance strategies.^17^, ^18^

Considering the scarcity of controlled studies in vestibular migraine and that posturography with virtual reality analyzes the effects of visual, somatosensory and vestibular stimuli on body balance maintenance, the interest in assessing the usefulness of this instrument developed, aiming to identify signs of postural control alterations in patients with this complex condition, very frequently observed in the interval between vertiginous episodes. The hypothesis for this study is that patients with vestibular migraine, even during the intercritical period of the condition, may be unable to maintain postural control when submitted to visual, somatosensory and vestibular stimuli.

The aim of this investigation is to evaluate patients’ postural control at posturography with virtual reality in the intercritical period of vestibular migraine.

## Method

This descriptive and analytical cross-sectional study was carried out from 2016 to 2017 in the Otology and Neurotology Discipline of the Department of Otorhinolaryngology Head and Neck Surgery of Universidade Federal de São Paulo (UNIFESP), Escola Paulista de Medicina (EFM), after approval by the Research Ethics Committee of the institution under number 1,873,209. All participants received information about the study and its objectives through an explanatory letter and signed the Free and Informed Consent Form before the investigation started. For the evaluation of body balance, the sample consisted of an experimental group of patients, which was sequentially selected, with a diagnosis of vestibular migraine established by an otorhinolaryngologist, in the intercritical period of the condition, and by a control group consisting of healthy individuals with no previous personal history and family history of migraine, comprising community volunteers, homogeneous regarding age and gender in relation to the experimental group.

The inclusion criteria for the control subjects were: healthy individuals, without symptoms, personal or family history of vestibular migraine or neurological, vestibular, auditory or other chronic diseases that could lead to balance alterations. Individuals who did not meet the inclusion criteria of this study were excluded from the research protocol.

The inclusion criterion for the experimental group consisted of male and female patients, between 18 and 64 years of age, with a diagnosis of vestibular migraine, who were selected from the vestibular migraine outpatient clinic^2^ during the intercritical period of the disease. The exclusion criteria for patients with vestibular migraine were: presenting other neurological disorders, other labyrinth diseases, inability to understand and follow simple verbal commands, reduced visual acuity that could interfere with the visualization of the test stimuli, inability to independently remain in the orthostatic position, orthopedic lower limb disorders that restrict movement, amputation or users of lower-limb prostheses, and use of medications with an effect on the vestibular system.

Patients from the experimental group underwent otorhinolaryngological examination; anamnesis; were submitted to the Brazilian version^19^ of the Dizziness Handicap Inventory (DHI);^20^ visual analog scale of vertigo and/or dizziness;^21^ and static posturography using the Balance Rehabilitation Unit (BRU™), Medicaa/Interacoustics, Uruguay.^22^

The DHI evaluated the impact of dizziness on the patient's quality of life. The DHI consists of twenty-five questions; seven on physical, nine on emotional, and nine on functional aspects. In each question, the patients answered yes, sometimes or no, corresponding to 4, 2 or 0 (zero) points, respectively. The total score ranges from (0) zero to 100 points, having 28 points as the maximum score for the physical aspect questions, 36 points for the emotional aspect and 36 points for the functional aspect. The DHI analysis classified the impact of symptoms on quality of life as mild, when the score was between (0) zero and 30, moderate between 31 and 60 and severe between 61 and 100 points.^23^

The visual analog scale of vertigo and/or dizziness measured the intensity of these symptoms to quantify the degree of patient discomfort, through a score ranging from (0) zero to 10 on a scale, with 0 (zero) being the lowest intensity and 10 (ten) the highest intensity of symptom level.^21^

The experimental and the control groups underwent body balance evaluation through the posturography module integrated to visual stimuli, projected in BRU™ virtual reality goggles. This posturography test evaluates the interaction between the visual, somatosensory and vestibular systems, measures postural sway resulting from exposure to different conditions on firm ground, on unstable surface with open eyes and closed eyes; and, visual stimulation, with or without head movements, that generates saccadic, optokinetic, vestibuloocular and vestibulospinal reflexes. The available visual stimuli, according to the calibrated oculomotor reflex, are: foveal (smooth pursuit, saccadic), retinal (linear optokinetic bars) and sensory interaction (optokinetic bars associated with the vestibular-ocular reflex to vertical and horizontal head movements).^22^

The equipment includes a computer with the BRU™ program; metallic safety structure; protector bracket with straps and seat belt; fixed force platform; virtual reality googles (eMagin Z800 3D Vision, eMagin, New York); accelerometer and unstable polyurethane foam surface with 14 cm-high density.^22^ The platform measures 40 × 40 cm, marked by coordinates displayed at its center, formed by a latero-lateral 8-cm line (intermaleolar line) and a 12-cm antero-posterior line that intercepts the midpoint of the intermaleolar line, to guide the adequate positioning of the individual's feet.

The posturography performed with the BRU™ provided information on the individual's pressure center position through quantitative indicators: limit of the stability area, pressure center area and sway velocity under ten sensory conditions. The limit of the stability area was measured by approximation of the sway pattern of an ellipse using the maximum and minimum total displacements to the right, to the left, forward and backward. The pressure center area was defined as the area of distribution of 95% of the samples of the pressure center; and the mean sway velocity was established by the total distance covered by the pressure center and divided by the time of 60 s of the test.^22^

In a quiet room measuring approximately six square meters, the test was performed with the patient standing upright on the platform, barefoot, arms extended along the body, with the right and left internal malleoli positioned at the extremities of the intermaleolar line and the two first toes 10° apart from the midline.

A foot mold^24^ helped to establish this angle. The midpoint of the intermaleolar line was used as the center of the standard limit of the stability circle.

The mean time for the complete evaluation with the BRU™was approximately 11 min, with 60 s for each situation, except for establishing the limit of stability, in which the sequence of movements can be completed before completing this period of time. Each individual had a personalized interval between the tasks, depending on their condition at the end of each one, whether they felt dizzy or not, or asked questions. The patient’s safety, regarding the risk of a possible fall, was ensured by the use of a protector bracket with straps and a safety belt.

To determine the limit of stability, the individual was instructed to perform maximal anteroposterior and lateral body displacements using the ankle strategy, without moving the feet or using trunk strategies. The patients moved slowly until they reached their body limit of stability according to the following sequence: 1) forward; 2) return to starting position; 3) to the right; 4) return to starting position; 5) to the left; 6) return to starting position; 7) backward; and 8) return to the starting position. The patients were asked to perform this sequence of movements twice, within the 60 s stipulated for this evaluation.^22^ The procedure was restarted in cases where the patient moved the feet or the trunk.

To evaluate the pressure center area and the sway velocity in ten sensory conditions, the patient was instructed to remain still in orthostatic position for 60 s: 1) on firm ground, with open eyes; 2) on firm ground, with closed eyes; 3) on a foam pillow, with closed eyes; 4) on firm ground, with saccadic stimulation; 5) on firm ground, with optokinetic stimulation in the horizontal direction, from left to right; 6) on firm ground, with optokinetic stimulation in the horizontal direction, from right to left; 7) on firm ground, with optokinetic stimulation in the vertical direction, from top to bottom; 8) on firm ground, with optokinetic stimulation in the vertical direction, from the bottom up; 9) on firm ground, with optokinetic stimulation in the horizontal direction, associated with slow and uniform head rotation movements; 10) on firm ground, with optokinetic stimulation in the vertical direction, associated with slow and uniform head flexion and extension movements.

The virtual reality googles were used in the evaluations from the 4^th^ to the 10^th^ conditions, along with a mask, to occlude the lateral view. The individuals were instructed in the fourth condition to follow with their eyes randomized targets with different colors and letters, to stimulate the saccadic system; from the fifth to the eighth conditions, to look at the center of their visual field, at alternating black and white moving bands to induce optokinetic nystagmus; and for the ninth and tenth conditions, to look at the center of their visual field, at alternating black and white moving bars and to perform slow and uniform movements of rotation and flexion-extension of the head with steady shoulders and trunk, to elicit optokinetic nystagmus and vestibuloocular reflex.^22^

At the end of the evaluation, the platform software provided a worksheet with the values of the limit of stability area, pressure center area, and sway velocity.

The statistical analysis of the collected data was initially performed in a descriptive way through the mean, median, minimum and maximum values, standard deviation, absolute and relative frequencies (percentage). The inferential analyses used to confirm or disprove the findings of the descriptive analysis were: Mann–Whitney test in the comparison of the groups (control and experimental) according to age, pressure center area and sway velocity; Fisher's exac*t*-test in the comparison of the groups (control and experimental) according to the gender; Student’s *t* test for independent samples in the comparison of groups (control and experimental) according to the limit of stability area. The significance level was set at 5% (α = 0.05) in all the conclusions obtained through the inferential analyses. The data were entered into Excel 2010 for Windows spreadsheets for adequate storage of the information. The statistical analyses were performed using the SPSS statistical program SPSS (Statistical Package for the Social Sciences, version 25.0, IBM Corp., Armonk, NY, USA).

## Results

Body balance data were collected through the posturography evaluation of the BRU™ of patients with vestibular migraine, referred by the Outpatient Clinic of the Otology and Neurotology Discipline of the Department of Otorhinolaryngology Head and Neck Surgery of Universidade Federal de São Paulo (UNIFESP), Escola Paulista de Medicina (EPM).

The selected sample consisted of 56 individuals, 26 (47.16%) in the experimental group, with vestibular migraine in the intercritical period of the disease, and 30 (53.57%) in the control group. The group of patients with vestibular migraine comprised 25 (96.15%) women and 1 (3.85%) man. There was no significant difference between the groups regarding gender (*p* > 0.999).

The experimental group had a mean age of 41.15 ± 15.14 years, ranging from 17 to 68 years, whereas the control group had a mean age of 38.53 ± 16.37 years, ranging from 18 to 72 years. There was no significant difference between the groups in relation to age (*p* = 0.544).

Regarding the intensity of vertigo and/or dizziness, the experimental group had a mean score of 3.08 points (Standard Deviation - SD = 3.04), with a minimum value of 2 and a maximum value of 10.

The group with vestibular migraine had a mean score of 37.07 points as the total score (SD = 18.49) when the DHI quality of life questionnaire was applied, 14.00 points (SD = 6.04) for the physical aspect, 12.75 points (SD = 8.38) for the functional aspect, and 11.50 points (SD = 8.00) for the emotional aspect. All the cases from the two groups completed the evaluation of the ten sensory conditions at the posturography with the BRU™, without moving the feet and upper limbs or falling.

**Figure 1:**
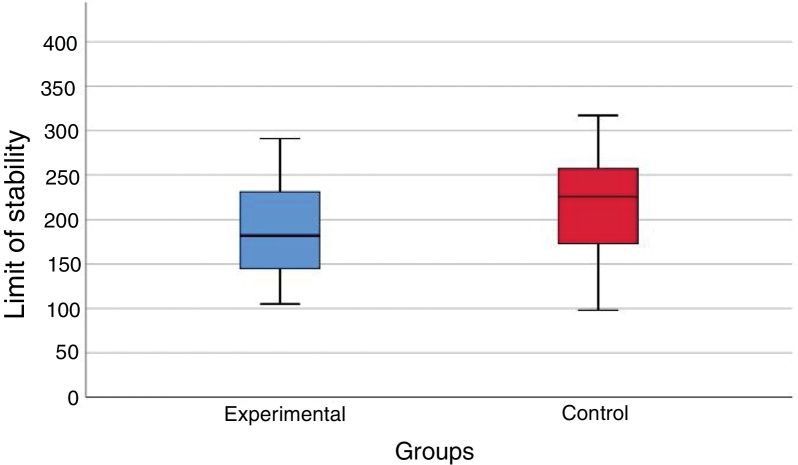
Graphic representation of the limit of stability area values (cm^2^) in the vestibular migraine group (experimental) and the control group in the Balance Rehabilitation Unit (BRU™) (*p* = 0.102).

At the posturography evaluation with the BRU™, there was no statistically significant difference (*p* = 0.102) between the values of the limit of stability area (cm^2^) in the vestibular migraine group and the values obtained in the control group (Fig. 1).

**Figure 2:**
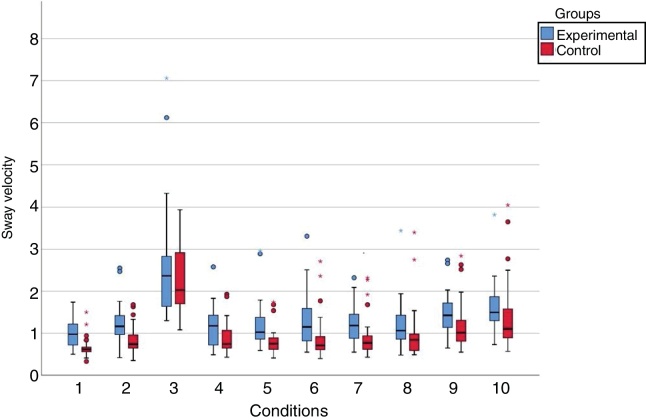
Graphic representation of the sway velocity values (cm/s) of the group with vestibular migraine and the control group at the Balance Rehabilitation Unit (BRU™). Conditions: 1) On firm ground, with open eyes (*p* < 0.001*); 2) On firm ground, with closed eyes (*p* = 0.002*); 3) On a foam pillow, with closed eyes (*p* = 0.341); 4) On firm ground, with saccadic stimulation (*p* = 0.026*); 5) On firm ground, with optokinetic stimulation in the horizontal direction, from left to right (*p* < 0.001*); 6) On firm ground, with optokinetic stimulation in the horizontal direction, from right to left (*p* = 0.001*); 7) On firm ground, with optokinetic stimulation in the vertical direction, from top to bottom (*p* = 0.003*); 8) On firm ground, with optokinetic stimulation in the vertical direction, from the bottom up (*p* = 0.007*); 9) On firm ground, with optokinetic stimulation in the horizontal direction, associated with slow and uniform head rotation movements (*p* = 0.036*); 10) On firm ground, with optokinetic stimulation in the vertical direction associated with slow and uniform head flexion and extension movements (*p* = 0.028*).

The mean values of the sway velocity (cm/s) in the experimental group were higher than those in the control group in the ten assessed conditions. When comparing the experimental and the control groups, statistically significant differences (*p* < 0.05) were observed between the sway velocity values in nine of the ten sensory conditions; there was no significant difference for the unstable surface condition and closed eyes (*p* = 0.341) (Fig. 2).

**Figure 3:**
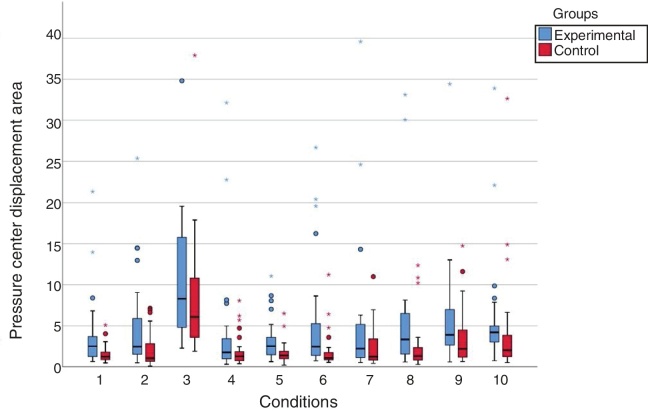
Graphic representation of the values of the pressure center displacement area (cm/s) of the group with vestibular migraine and the control group at the Balance Rehabilitation Unit (BRU™). Conditions: 1) On firm ground, with open eyes (*p* = 0.003*); 2) On firm ground, with closed eyes (*p* = 0.003*); 3) On a foam pillow, with closed eyes (*p* = 0.139); 4) On firm ground, with saccadic stimulation (*p* = 0.066); 5) On firm ground, with optokinetic stimulation in the horizontal direction, from left to right (*p* = 0.002*); 6) On firm ground, with optokinetic stimulation in the horizontal direction, from right to left (*p* = 0.002*); 7) On firm ground, with optokinetic stimulation in the vertical direction, from top to bottom (*p* = 0.022*); 8) On firm ground, with optokinetic stimulation in the vertical direction, from the bottom up (*p* = 0.007*); 9) On firm ground, with optokinetic stimulation in the horizontal direction, associated with slow and uniform head rotation movements (*p* = 0.027*); 10) On firm ground, with optokinetic stimulation in the vertical direction, associated with slow and uniform head flexion and extension movements (*p* = 0.003*).

The mean values of the pressure center displacement area (cm^2^) in the experimental group were higher than those of the control group in the ten assessed conditions. There was a significant difference (*p* < 0.05) between the values of the pressure center displacement area in eight of the ten sensory conditions; no significant difference was found for the condition unstable surface and closed eyes (*p* = 0.139) and for the condition on firm ground and saccadic stimulation (*p* = 0.066) (Fig. 3).

## Discussion

The static posturography integrated to visual stimuli of the BRU™ virtual reality system evaluated the body balance of patients in the intercritical period of vestibular migraine in comparison to a group of healthy individuals. In vestibular disorders and healthy young and elderly individuals, the reliability and validity of pressure center measurements obtained during the evaluation of the sensory integration processes were demonstrated using the BRU™, showing clinical validity and efficacy similar to those of the SMART EquiTestTM sensory organization test (NeuroCom Inc., Clackamas, USA).^25^ No literature citations were found on the limit of stability or changes in the sway velocity and the pressure center displacement area in patients with vestibular migraine at the static posturography using the BRU™, highlighting the originality of this investigation.

The groups were homogeneous in relation to age and gender, with a predominance of females. Vestibular migraine occurs more frequently in women, at any age group,^1^ similar to this study sample.

The influence of the symptoms was classified as mild^21^ after the analog scale of vertigo and/or dizziness was applied and the impairment in the patients' quality of life was characterized as moderate^23^ after the Brazilian version^19^ of DHI was applied,^20^ corroborating the report of relevant effects of the disorder on the quality of life.^3^

During the static posturography assessment using the BRU™, the limit of the stability area, similar in the patients during the intercritical period of vestibular migraine and in the control group, showed the preservation of the capacity to safely move the body to the maximum in the anteroposterior and latero-lateral direction on the pressure center without changing the support base.

At the static posturography with the BRU™ in vestibular migraine, in comparison with the control group, the increase in the sway velocity in nine of the ten conditions and in the pressure center displacement area in eight conditions demonstrated the objective postural instability of the patients with vestibular migraine, even in the intercritical period of the disorder, suggesting the influence of the vision, proprioception, visual stimuli and vestibulovisual interaction on postural control and identified impairments in the ability to effectively use vestibular, visual and somatosensory information.

The increase in the sway velocity and/or the pressure center displacement area in vestibular migraine was observed under conditions 1, 2, 5, 6, 7, 8, 9 and 10. Condition 1, on firm ground with open eyes, indicated that the use of visual and somatosensory cues was insufficient to compensate for the inaccurate vestibular system information. Condition 2, on firm ground and closed eyes, indicated that the somatosensory and vestibular cues were not sufficient without the visual ones. Conditions 5–8, on firm ground and optokinetic stimulation to the right, to the left, upward and downward, showed the inability to suppress the influence of the optokinetic visual information and rely on proprioceptive and vestibular stimuli, characterizing visual dependence.^26^ Conditions 9 and 10, on firm ground and horizontal and vertical vestibular-visual interaction, suggested the impairment of the fusion mechanism of visual and vestibular information, which is important for the stabilization of the image in the retina and to maintain spatial orientation and postural stability. The vestibuloocular reflexes, triggered by the head movements, and the optokinetic oculomotor reflexes, generated by the velocity of visual flow, must be adapted so that the image remains stable in the retina; any change in the gain of these reflexes or joint processing in the central nervous system, results in the instability of the image in the retina and reweighting of information, readjustment of the reflex gain and the introduction of other compensatory reflexes, such as the vestibulocollic reflex, among others, to obtain image stability.^27^

The patients with vestibular migraine assessed by the BRU™ showed similar performance at the sway velocity and the pressure center displacement area control in Condition 3, on unstable surface and closed eyes, the most challenging of the conditions, in which the somatosensory cue is imprecise on the foam pillow and visual information is absent, and the vestibular system is the main source of sensory information to maintain postural control.

The difficulty in standing upright with eyes closed on an unstable surface was also identified in 31.8% of individuals aged 40 years or older without a history of dizziness^28^ and in patients with vestibular migraine compared to healthy volunteers at the Tetrax IBS™,^14^, ^15^ corroborating the finding of the present study. Therefore, the patients with vestibular migraine did not show lack of postural control with unavailable visual cues and altered somatosensory signals or the posturography using the BRU™ was unable to identify vestibular impairment in this condition.

The patients with vestibular migraine assessed at the BRU™ showed, in Condition 4, on firm ground and saccadic stimulation, a pressure center displacement area similar to that of the control group; the lower body sway during exposure to saccadic stimulation would facilitate the control of ocular movements and target visualization.^29^

Therefore, considering these findings, the patterns identified in the group of patients with vestibular migraine included in this study corresponded to: vestibular dysfunction (Condition 1), vestibular and somatosensory dysfunction with visual dependence (Conditions 2, 4, 5, 6, 7 and 8) and vestibulovisual dysfunction (9 and 10).

It is difficult to compare the findings in the intercritical period of vestibular migraine in the virtual reality posturography using the BRU™ with those of other types of posturography that use different technologies, protocols and evaluation parameters. However, results from other types of posturography related to the capacity to maintain postural control also show the increased sway in some sensory conditions in vestibular migraine, suggesting the difficulty to use vestibular information with or without somatosensory and/or visual dependence.^4^, ^11^, ^12^, ^13^, ^14^, ^15^

The pressure center displacement area and sway velocity increased at the BRU™, showing worse postural control with and without visual deprivation, due to visual conflict and vestibulovisual interaction, suggesting that functional alterations may persist even in the asymptomatic intercritical period of the vestibular migraine, similarly to what occurs in other vestibular disorders, such as Meniere's disease and Benign Paroxysmal Positional Vertigo (BPPV). In Menière’s disease^30^ and in BPPV,^31^ which are very frequent vestibulopathies, alterations in the pressure center displacement area and in the sway velocity at the BRU™ were also found in several sensory conditions, demonstrating the involvement of the vestibular, visual and proprioceptive pathways.

The identification of significant findings in the intercritical period of vestibular migraine, when compared to healthy subjects, showed the clinical applicability of posturography integrated to the virtual reality of the BRU™ system, recreating environmental stimuli and situations capable of measuring postural responses to different sensory conditions, sensitizing the semiological exploration and disclosing relevant information on body balance.

The characterization of the postural control disorder, even during the interval between vertiginous episodes, can provide valuable data on the patient's functional capacity, assisting in the early diagnosis, prevention of the potential occurrence of symptoms resulting from dysfunction signs identified before the clinical episode, and in the creation of personalized protocols of vestibular rehabilitation exercises. New experiments will be useful to continue investigating postural control in vestibular migraine and to confirm the findings of this research.

## Conclusion

Posturography with virtual reality can identify changes in the sway velocity and the pressure center displacement area, characterizing the inability to maintain postural control with and without visual deprivation, in situations of visual conflict and vestibulovisual interaction, in the intercritical period of vestibular migraine.

## Conflicts of interest

The authors declare no conflicts of interest.
